# Chaos-Embedded Multi-Objective Intelligent Optimization-Based Explainable Classification Model for Determining Cherry Fruit Fly Infestation Levels Using Pomological Data

**DOI:** 10.3390/biomimetics11030218

**Published:** 2026-03-18

**Authors:** Suna Yildirim, Inanc Ozgen, Bilal Alatas, Hakan Yildirim

**Affiliations:** 1 Department of Software Engineering, Malatya Turgut Özal University, 44210 Malatya, Türkiye; suna.yildirim@ozal.edu.tr; 2 Department of Bioengineering, Firat University, 23119 Elazig, Türkiye; 3 Department of Software Engineering, Firat University, 23119 Elazig, Türkiye; balatas@firat.edu.tr; 4 Department of Horticulture, Malatya Turgut Özal University, 44210 Malatya, Türkiye; hakan.yildirim@ozal.edu.tr

**Keywords:** *Rhagoletis cerasi*, pomological traits, evolutionary algorithm, explainable AI, pest population classification

## Abstract

The European cherry fruit fly (*Rhagoletis cerasi* L.) poses a significant pest threat to cherry production due to its rapid reproduction and host specificity, causing substantial economic damage. This study presents a novel, explainable, and biologically inspired data-driven classification model based on fruit characteristics to support targeted and sustainable pest control strategies. In research conducted at four different locations in Elazığ province, three population classes were determined based on the number of adult individuals caught in traps, and 10 different fruit characteristics were measured in fruit samples belonging to each class. The data used in this study are original data obtained by the authors. To examine the relationship between pomological characteristics of cherry fruit and cherry fruit fly density, the Chaotic Rule-based–Strength Pareto Evolutionary Algorithm2 (CRb-SPEA2) method, developed as a multi-objective and chaos-integrated evolutionary rule mining framework, was adapted. The developed algorithm aimed for high performance, interpretability, and transparency. Accuracy, Precision, and Recall metrics, which are conflicting objectives, were optimized with Pareto-optimal solutions, yielding selectable results for domain experts. To increase population diversity and reduce the risk of early convergence and getting stuck in a local optimum, the Tent chaotic mapping mechanism was also integrated into the system. Furthermore, the model was trained without the need for predefined automatic discretization of the continuous value ranges of the attributes. The proposed model achieved superior results across all classes, with the highest accuracy rate of 82.6% recorded in the High class, demonstrating excellent sensitivity and recall values.

## 1. Introduction

Global cherry production is approximately 2.4 million tons, with Turkey leading the world in cherry production, accounting for 627,000 tons and a 26% global share [1]. Sweet cherry (*Prunus avium* L.) is considered a high-value economic fruit crop in temperate regions due to its strong market demand, superior organoleptic quality, and wide use in both fresh and processed forms [2]. In addition to its economic relevance, cherries are rich in vitamins, minerals, and phenolic antioxidants, particularly anthocyanins, which have been associated with various health benefits and increased consumer preference [3]. These economic and nutritional attributes have led to a continuous expansion of cherry cultivation areas worldwide, including Türkiye. While cherries are cultivated across a broad geographical range worldwide, major commercial production occurs in countries such as Turkey, the United States, Iran, and Italy. In Turkey, cherry cultivation occupies a wide production area and continues to expand as the number of trees increases annually. However, several pests and diseases significantly limit cherry yield, among which the cherry fruit fly, *Rhagoletis cerasi* L. (Diptera: Tephritidae), plays a critical role [4,5]. The timing of chemical control interventions is closely tied to key phenological stages of the cherry, particularly flowering, fruit set, and maturation. According to integrated pest management guidelines, the onset of fruit coloring (pink stage) is a key indicator for initiating chemical treatments [4]. Yet, this timing is influenced by cultivar differences and local meteorological conditions. Over the past decade, chemical pesticides have become the predominant control method for *R. cerasi*, largely due to their ease of application, rapid results, and widespread market availability [2]. However, extensive use of such chemicals poses risks to human health, biodiversity, and the environment, including soil and water contamination, development of resistance, and economic losses [4,6,7].

An alternative approach gaining traction is biotechnical control, which includes the use of yellow sticky traps containing ammonium capsules for both monitoring and mass trapping of adult flies [8,9]. Especially when combined with pheromones, these traps can be deployed extensively to achieve effective population reduction. Due to their species-specific action and minimal side effects, biotechnical methods are increasingly adopted worldwide [10,11]. In Elazig Province, such traps have been actively used in recent years, with studies investigating the influence of variables like light intensity and elevation on pest population dynamics [12,13]. Certain pomological traits of cherry fruits such as color, weight, width, length, stem length, firmness, pit weight, soluble solid content (SSC), ripeness level determined by NaOH, and acidity not only determine fruit quality but also influence the host selection behavior of fruit flies [14]. Specifically, *R. cerasi* and related species are known to be highly sensitive to both the physical and chemical properties of the host fruit during oviposition [15]. Nature-inspired optimization algorithms, which mimic biological growth and adaptation mechanisms, have become increasingly popular for solving complex real-world problems. Priyadarshini introduced Dendritic Growth Optimization (DGO), an algorithm inspired by natural branching patterns, and argued that it yielded successful results when compared to classical machine learning algorithms [16].

Recent studies have aimed to develop classification models based on pomological data, particularly across five distinct fruit coloring stages. Among these efforts, three evolutionary algorithms, Evolutionary Rule Extractor for Classification (CORE), Data Mining with Evolutionary Learning for Classification (DMEL) and Organizational Evolutionary Classification (OCEC), have been used to extract interpretable rules for identifying whether a given fruit sample belongs to the second pomological period, known to coincide with peak *R. cerasi* activity in the region [17]. Recent advances in multi-objective evolutionary optimization have explored integrating domain knowledge into evolutionary search processes. [18] proposed a process knowledge-guided autonomous evolutionary optimization framework for constrained multi-objective problems that dynamically adapts search strategies based on population evolution patterns and constraint characteristics, improving convergence efficiency and robustness. In addition, [19] introduced a population image convolution mechanism that enhances diversity maintenance and exploration capability by analyzing population distribution characteristics in high-dimensional optimization spaces. However, these studies did not incorporate pest population data and therefore failed to explore the relationship between fruit ripeness stages and pest density. To address this gap, the present study introduces a novel classification approach based on pomological data that also integrates cherry fruit fly population levels. The dataset was categorized into three pest population levels, Low (L), Medium (M), and High (H), based on the number of adults captured in traps. To perform this classification, a multi-objective evolutionary algorithm, CRb-SPEA2, was employed [20]. The primary motivation behind this study is to model the complex and nonlinear relationships frequently encountered in agricultural data mining through a transparent and interpretable model. Unlike evolutionary optimization algorithms, the proposed CRb-SPEA2 algorithm simultaneously optimizes performance metrics such as accuracy, precision, and recall. These metrics provide expert decision-makers with a set of solutions to choose from depending on the situation. The proposed method generates rule sets by optimizing the dynamic range interactions between attributes such as fruit color, acidity, and hardness in the dataset. The algorithm overcomes the handicap of getting stuck in local optima by using Tent chaotic mapping to achieve global solutions. One of the strong distinguishing features of our method is that, unlike the black-box structure of traditional methods, it provides explainable, transparent solutions and understandable if–then rules. These advantages offer field experts a practical and scientifically valid decision support mechanism. The present study makes an original contribution to cherry fruit fly management by establishing, for the first time, a direct and explainable link between pomological characteristics of cherry fruits and actual *Rhagoletis cerasi* population density. Unlike previous studies that focused either on phenology-based calendar recommendations or image-based black-box detection models, this research integrates field-derived population data with fruit quality parameters and analyzes them through a multi-objective evolutionary algorithm. The proposed CRb-SPEA2 model generates transparent if–then rules that reveal which combinations of fruit color, firmness, acidity, and maturity indicators correspond to low, medium, or high infestation risk. This approach provides growers and decision makers with a biologically interpretable decision-support tool that enables optimization of control timing, reduction in unnecessary pesticide applications, and promotion of environmentally sustainable cherry production.

## 2. Materials and Methods

**Figure 1:**
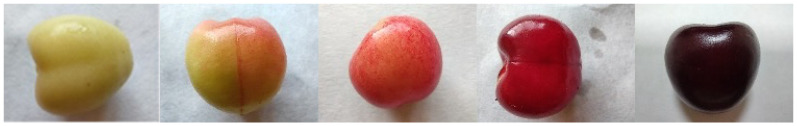
Cherry fruits collected at five different developmental stages used for determining pomological characteristics. The samples represent ripening scales (Scale 1–Scale 5) collected during harvest and used to obtain the pomological measurements forming the dataset used in the experiments.

In this study, the cherry cultivar Ziraat 900, which is commonly grown in the region for commercial purposes, was used. The fruits of this cultivar served as the primary material for the research. The study began on 1 March 2024, and starting from April 1, 2024, yellow sticky traps with ammonium capsules were placed in the orchards. The number of adult cherry fruit flies caught in the traps was recorded at 5 to 7 day intervals. These counts continued until 22 June 2024, and within ten days following that date, the cherry fruits were harvested. A total of four different locations (Harput 1, Harput 2, Baskil 1, and Baskil 2) were included in the study, each cultivating the same Ziraat 900 variety across five-decare orchards. In each orchard, five traps were installed, and adult fly counts were recorded during different fruit coloring periods (Figure 1). In the study, cherry fruit fly numbers captured using yellow sticky traps were analyzed as a multi-objective rule mining problems. In total, the dataset consisted of 381 samples collected from the four orchard locations. The samples were categorized into three infestation classes based on the number of adult flies captured in the traps: Low (L), Medium (M), and High (H). The class distribution included 204 samples for the Low class, 101 samples for the Medium class, and 76 samples for the High class. Although the dataset shows a moderate class imbalance, this distribution reflects the natural population dynamics of cherry fruit fly observed under field conditions. Collecting samples from multiple orchard locations also improves the representativeness of the dataset by capturing spatial variability in pomological characteristics and pest population levels. Adult fly numbers in the traps were monitored weekly (sometimes every 5 days) and this continued until the end of the season. This time-extended monitoring allowed for observation of pest changes during the phenological stages of fruit development. Data from trees in the same orchard were also used to examine the impact of the changing pomological profile as the fruit matured on the classification. Samples of five different colored fruits were collected and transported to the laboratory using a cold chain. Both physical and chemical indicators were evaluated for each fruit sample. Thus, classification considered all attributes of the fruit, rather than relying on a single attribute. The rules derived from the experiments are of an if–then structure, with each rule representing a single individual. To measure the success of the model, not only the accuracy value was taken, but also the precision and recall metrics were calculated, optimizing multiple objectives simultaneously. The problem of getting stuck in local optima during optimization processes is prevented by the Tent Chaotic Mapping mechanism integrated into the algorithm. During the algorithm, the non-dominated individuals obtained in each iteration are stored in an external archive, and the numerical value ranges of the fruit’s attributes are optimized using genetic operators. The rules obtained as a result of these steps produce interpretable results for subject matter experts, unlike the black-box nature of classical machine learning methods.

Cherry fruits from different developmental stages were collected to determine their pomological characteristics. During the harvest period, samples were taken from the same trees on which the traps had been placed, specifically on 25 April 2024 (Scale 1), 14 May 2024 (Scale 2), 22 May 2024 (Scale 3), 27 May 2024 (Scale 4), and 7 June 2024 (Scale 5). These samples, representing different ripening stages (Figure 1), were collected from four locations in Elazig Province Harput 1, Harput 2, Baskil 1, and Baskil 2, and then stored under refrigeration and later sent to the Malatya Fruit Research Institute for analysis. In these analyses, various pomological parameters were measured for each location, including fruit weight, width, length, height, stem length, fruit firmness, pit weight, soluble solid content (SSC), NaOH-based ripeness, and acidity levels. The obtained data were subsequently used for classification purposes. The characteristics of the pomological dataset utilized in the experiments in this study are provided in Table 1. The data contained within the dataset were then subjected to classification as Low (L), Medium (M), or High (H) classes, with this classification being determined by the number of adults captured in the traps. The value ranges for class determination were set as 0–5 for Low, 6–10 for Medium, and 10 or more for High classes. The total number of datasets was 381, with 204 data classified as L, 101 as M, and 76 as H. Although the dataset contains a limited number of samples (381 instances), rule-based evolutionary learning approaches such as CRb-SPEA2 focus on extracting interpretable classification rules rather than fitting highly parameterized models. This characteristic helps reduce the risk of severe overfitting. Furthermore, the model performance was evaluated using multiple metrics (Accuracy, Precision, and Recall) within a multi-objective optimization framework, which promotes balanced and generalizable solutions.

### 2.1. CRb-SPEA2 Algorithm

The CRb-SPEA2 algorithm, which was developed by the authors, was utilized in the experiments [20]. The CRb-SPEA2 algorithm is based on the SPEA2 [21] algorithm, and the selection of an appropriate representation format and the evaluation of the extracted rules according to the objective values are added. Moreover, the developed method is not a black-box method and offers explainability. The SPEA2 algorithm employs the Pareto-front approach when confronted with conflicting objectives. The objective is to attain a balanced solution space and accelerated convergence. The SPEA2 algorithm commences the optimization process with an empty archive and an initial population. The utilization of strength and raw fitness values serves as a metric for the assessment of competitive dominance among individuals. The Strength (*S*(*i*)) value is indicative of the number of individuals dominated by the i-th individual at time t (Equation (1)). The Raw Fitness (*R*(*i*)) value is indicative of the number of times the individual has been dominated. It can be demonstrated that the closer this value is to 0, the less dominated it is (Equation (2)).

(1) $$S\left(i\right)=\left|\left\{\left.j\right|j\in P_{t}+{\bar{P}}_{t}⋀i≻j\right\}\right|$$

(2) $$R\left(i\right)=\sum_{j\in P_{t}+\bar{P_{t}},j≻i}St(i)$$

In many cases, the raw fitness value may not provide complete information about a candidate’s dominance. In order to address this deficiency, the density value (*D*(*i*)) is added to the algorithm using the k-Nearest Neighbor method (kNN). The objective function is thus calculated as indicated in Equation (3).

(3) $$F\left(i\right)=R\left(i\right)+D\left(i\right)$$

Based on the fitness function, candidates with a fitness value less than 1 are selected to move on to the next archive. This process is repeated until the archive size is reached. Furthermore, the Tent chaotic map was used to create a randomization mechanism in the CRb-SPEA2 algorithm [20] (Equation (4)). Chaotic maps are frequently used in optimization problems to improve exploration capabilities during the search process. In this study, a Tent Chaotic map was used in the initialization phase of the CRb-SPEA2 algorithm to create a homogeneously distributed initial population and increase diversity. Increasing population diversity also reduced the probability of early convergence during evolutionary search. The Tent map generates a chaotic sequence *X_n_* within the interval (0,1). *X_n_* represents the chaotic sequence in the *n*-th iteration, while ∂ is the control parameter of the chaotic system. In this study, ∂ = 2 was used to ensure fully developed chaotic behavior and homogeneity of the search space. Nevertheless, future studies may include a detailed sensitivity analysis of the chaotic map parameters, such as the initial value and bifurcation coefficient, in order to further investigate their influence on convergence behavior and solution diversity. The pseudo code for proposed CRb-SPEA2 algorithm is presented in Algorithm 1.

(4) $$X_{n+1}=\left\{\begin{array}{ll} \partial X_{n}, & X_{n}<\frac{1}{2}\\ \partial \left(1-X_{n}\right), & \frac{1}{2}\leq X_{n} \end{array}\right\}$$

**Algorithm 1.** Pseudocode of the proposed CRb-SPEA2 multi-objective evolutionary rule mining algorithm.Input: Pomological Dataset, Population Size (*N*), Archive Size ($\bar{N}$) Max Generations (*T*) Output: Pareto-optimal Rule Set
Chaotic Initialization:               Set an initial chaotic value $x_{0}\in (0,1)$.               For each individual, generate initial values using the Tent Chaotic Map.               Map the chaotic sequences to initialize rule thresholds for pomological features.
2.Fitness Evaluation:               Calculate Accuracy, Precision, and Recall for each rule.
3.Evolutionary Loop:              Environmental Selection: Update the Archive ($\bar{P}$) with non-dominated individuals.               Apply the truncation operator if the archive size exceeds ($\bar{N}$).               Mating Selection: Perform binary tournament selection from the Archive.               Variation: Apply crossover and Chaotic Mutation.               Update the population with new offspring.
4.Termination: Return the set of non-dominated rules from the Archive.

### 2.2. Rule Mining Model

The representation format used in the CRb-SPEA2 algorithm consists of three vectors. These vectors are ${(n}_{i}^{b})$ vector, which indicates whether the attribute will be included in the rule or not, which is determined by a predetermined threshold value, and ${(n}_{i}^{l})$ and ${(n}_{i}^{u})$ vectors, which indicate the lower and upper values of the relevant attribute (Equation (5)).

(5) $$a_{i}=\left\{\begin{array}{cc} 1\left(\mathrm{Feature}\;i\;\mathrm{is}\;\mathrm{used}\;\mathrm{in}\;\mathrm{the}\;\mathrm{rule}\right) & \mathrm{if}\;a_{i}^{b}>\lambda ,\;\lambda \;\epsilon \left[0,1\right]\\ 0 & \mathrm{otherwise} \end{array}\right.$$

A rule is generated for each iteration of the resulting candidate solutions, and this rule can change in subsequent iterations. If a rule contains attributes 4 and 8, the rule derived to represent attribute F and class C is shown as in Equation (5). As shown in Equation (6), the rules consist of “if” and “then” parts. To examine the consistency of the rules, conflicting Accuracy, Precision, and Recall values are calculated (Equations (7)–(9)). The True Positive (TP), True Negative (TN), False Positive (FP), and False Negative (FN) values used to obtain these values are also calculated as in Table 2.

(6) $$if\;a_{4}^{l}\leq F_{4}\leq a_{4}^{u}\;and\;a_{8}^{l}\leq F_{8}\leq a_{8}^{u}\;then\;C$$

(7) $$Accuracy=\frac{TP+TN}{TP+TN+FP+FN}$$

(8) $$Precision=\frac{TP}{TP+FP}$$

(9) $$Recall=\frac{TP}{TP+FN}$$

**Figure 2:**
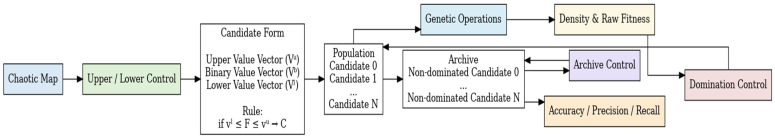
General mechanism of the proposed CRb-SPEA2 algorithm tailored for cherry fruit fly infestation classification. The flowchart illustrates the generation of candidate rules via chaotic mapping and their multi-objective evaluation based on accuracy, precision, and recall metrics. The process highlights how non-dominated rule sets are archived using Pareto dominance to ensure a balanced selection of explainable and high-performing solutions for pest level determination.

Figure 2 illustrates the general mechanism of the proposed CRb-SPEA2 algorithm. The basic structure is presented considering candidate representation, chaotic mapping, fitness and density functions, dominance control, and archive structure.

## 3. Results and Discussion

**Figure 3:**
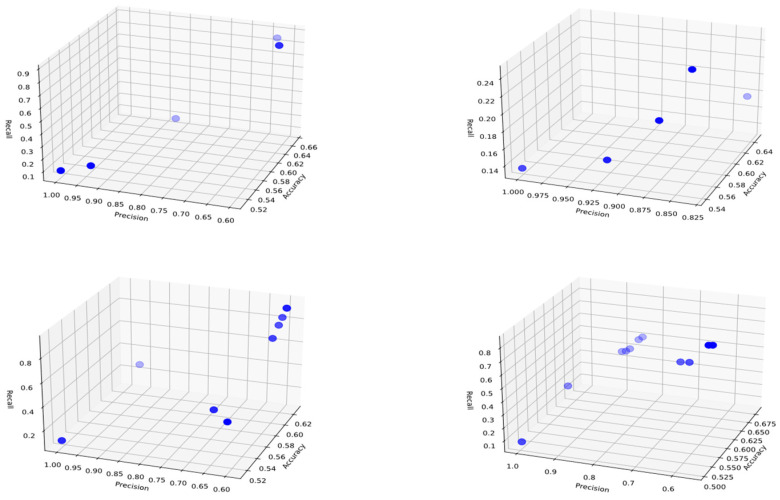
Three-dimensional Pareto charts representing the distribution of candidate rules for Class L during the training phase, derived from the pomological dataset of *Rhagoletis cerasi* L. Each point denotes a rule solution evaluated through the simultaneous optimization of accuracy, precision, and recall metrics. The charts demonstrate the multi-objective trade-off relationships across independent experimental runs, highlighting the model’s ability to identify robust and explainable rules for early-stage pest infestation levels.

**Figure 4:**
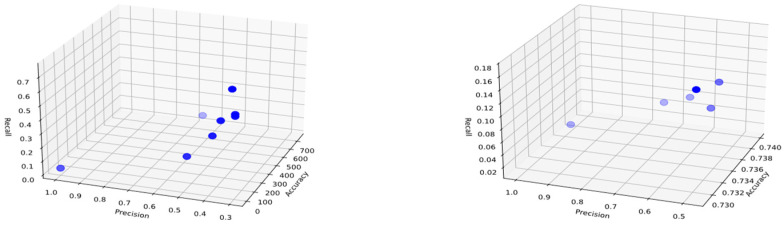

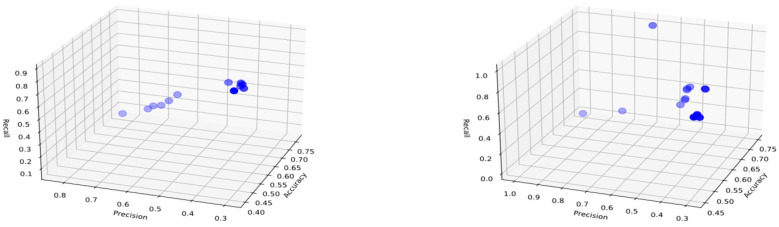
Three-dimensional Pareto charts representing the distribution of candidate rules for Class M during the training phase, derived from the pomological dataset of *Rhagoletis cerasi* L. Each point denotes a rule solution evaluated through the simultaneous optimization of accuracy, precision, and recall metrics. The charts demonstrate the multi-objective trade-off relationships across independent experimental runs, highlighting the model’s ability to identify robust and explainable rules for early-stage pest infestation levels.

**Figure 5:**
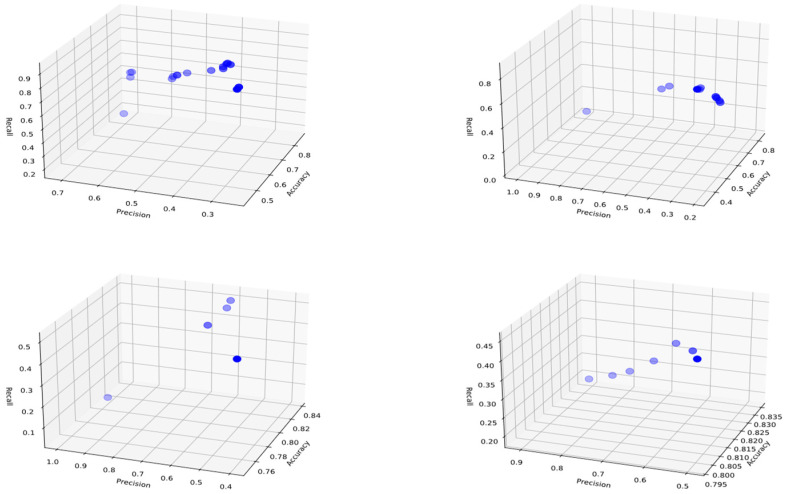
Three-dimensional Pareto charts representing the distribution of candidate rules for Class H during the training phase, derived from the pomological dataset of *Rhagoletis cerasi* L. Each point denotes a rule solution evaluated through the simultaneous optimization of accuracy, precision, and recall metrics. The charts demonstrate the multi-objective trade-off relationships across independent experimental runs, highlighting the model’s ability to identify robust and explainable rules for early-stage pest infestation levels.

Before conducting the rule inference process, the dataset was divided into two parts: 70% training data and 30% test data. 10 independent experiments were conducted for each class, with an initial population and archive size of 20. The threshold for including attributes in the rule for representation was set at 0.5, and the crossover and mutation probabilities were set at 0.8 and 0.1, respectively. As a result of the experiments, Pareto curves were generated for candidate solutions in each class during the training phase, and several examples are shown in Figure 3, Figure 4 and Figure 5. The graphs obtained using the Pareto-front-based CRb-SPEA2 algorithm simultaneously optimize and display conflicting accuracy, precision, and recall metrics in three-dimensional space. The points shown on the Pareto curves are ideal solution candidates, considered non-dominated and possessing their own rule set. These graphs, created separately for each class, show the solution sets produced by the algorithm at different population densities. When examining the Pareto curve of class H, it is observed that the candidate solutions cluster at higher values on the accuracy axis. This proves that the 82.6% accuracy value obtained in class H was not reached by chance and that the distinction between pomological features was well-made. The concept of diversity, another important issue in optimization, refers to the variety of solutions. Looking at the distribution of candidate solution points on the Pareto curves, it is seen that the algorithm does not focus on only one performance metric but also offers a trade-off between precision and recall metrics. The decision-maker has the freedom to choose the desired solution according to its needs from among multiple solutions presented. Early convergence, a drawback of optimization, was prevented by the Tent chaotic mapping system used, allowing for a broader search. Pareto curves, unlike those of classical machine learning methods, serve as proof that explainable and transparent solution sets are presented.

The distributions of Pareto optimal solutions in the objective space, showing the diversity of generated rule sets, are given in Figure 3, Figure 4 and Figure 5. Examination of these figures reveals that the candidate solutions are not concentrated at a single point but are located in different regions of the accuracy, precision, and recall axes. Pareto solutions for classes L and M exhibit a wider distribution, reflecting different balances between accuracy and recall values. In addition, solutions belonging to class H, while preserving variations in recall and precision, are concentrated in regions with higher accuracy. Thus, it is shown that the CRb-SPEA2 algorithm effectively explores the search space, generating multiple competing rules instead of a single optimal solution, thereby offering alternatives to decision-makers.

The model provides decision-makers with alternative solutions instead of a single one, thanks to the multiple Pareto-optimal rules obtained. In this study, the trade-off between accuracy, precision, and recall values allows field experts to select the appropriate rule based on their priorities. If the goal is to minimize false positive pesticide applications, high-precision rules can be chosen. If the aim is to minimize the risk of missing high infestation levels, rules with higher recall values can be preferred. These flexibilities allow experts to make practical decisions by selecting the most appropriate rule based on their pest monitoring strategies and management priorities, and by providing interpretable alternative solutions.

The rules generated for all classes and the statistical results for the Acc, Pre, and Rec values in both the training and testing phases are presented in Table 3, Table 4 and Table 5. When the mean and median values are considered in the statistical results, the lack of significant differences between them provides an idea of the optimization’s success. Table 3 shows that the rule sets automatically generated by our proposed CRb-SPEA2 algorithm correlated the determined physical and chemical characteristics of cherry fruit with *R. cerasi* population density, offering a threshold-based and applicable decision support mechanism that deviates from calendar-based approaches. In the study, parameters such as weight, width, length, height, stem length, fruit firmness, seed weight, SSC, NaOH-based maturity index, and acidity of each individual in the dataset were considered together, and the extent to which population growth is related to the maturity profile of the fruit was investigated. Looking at the structure of the rules, it is seen that in the L class, i.e., at the low population level, the risk is generally determined by early-stage fruit characteristics. The fact that pest pressure remains at a low level in periods where fruit weight is lower but acidity is high is also parallel to literature sources indicating that female flies prefer more mature periods for egg laying. In light of this information, if acidity values are taken as a basis, chemical applications can be postponed in stages where these values remain above a certain threshold, or the monitoring process can be extended and intervention delayed somewhat. When examining the rules developed for class M, it is observed that the risk of pests is not dependent on a single characteristic. Color, firmness, and NaOH levels are particularly decisive factors, especially during the pinkish stage when the cherry tissue begins to soften. In class H, which has a high population, pest symptoms are defined by multiple characteristic rules. Darkening of the fruit color, decrease in acidity, and increase in stem length and seed weight (indicating maturity) all indicate increased risk. Unlike black-box models, these if–then rules will assist decision-makers in identifying which changes in characteristics increase risk and determining the appropriate timing for chemical control.

In order to fulfill the requirement for a systematic analysis of decisive fruit characteristics, a feature importance study was performed. To better understand the contribution of pomological attributes to pest population classification, an additional feature importance analysis was conducted based on the frequency of attribute occurrence within the generated rules. Since the CRb-SPEA2 algorithm produces interpretable if–then rules, the relative importance of each feature can be evaluated by examining how frequently it appears in the rule sets associated with different infestation levels. The analysis revealed that certain fruit characteristics appear more consistently across the rule sets, indicating a stronger influence on the classification of cherry fruit fly population density. In particular, fruit color, acidity, NaOH-based maturity index, fruit weight, and width were among the most frequently observed attributes within the generated rules. These features represent key indicators of fruit maturity and physiological development, which are known to influence host selection behavior in *Rhagoletis cerasi*. When the rule structures are examined across the three infestation classes, distinct patterns emerge. For the Low population class (L), rules often include attributes such as higher acidity values and smaller fruit dimensions, reflecting early developmental stages of the fruit. This observation aligns with biological findings suggesting that female cherry fruit flies generally avoid oviposition in immature fruits. In the Medium population class (M), rule combinations frequently involve fruit color transitions, firmness, and NaOH-based maturity indicators, which correspond to intermediate ripening stages when fruit tissues begin to soften and become more suitable for oviposition. In contrast, the High population class (H) is typically characterized by rules involving darker fruit color, lower acidity levels, increased stem length, and higher seed weight, indicating advanced fruit maturity. These attributes suggest that the risk of infestation increases as the fruit approaches full ripeness. Overall, the rule frequency analysis confirms that the proposed model captures biologically meaningful relationships between pomological characteristics and pest population density. By identifying the most influential fruit attributes across infestation levels, the model provides interpretable insights that can assist growers and agricultural experts in determining critical stages for pest monitoring and control interventions.

In this part of the study, a comparison was made between the proposed method and classical machine learning (ML) methods (Table 6). In order to achieve this objective, the well-known algorithms of Multilayer Perceptron (MLP), Support Vector Machines (SVM), k-Nearest Neighbor (kNN/Instance-based kNN-IBk), and Naive Bayes (NB) were utilized. The experimental findings demonstrated that the Acc, Pre, and Rec values obtained from the CRb-SPEA2 algorithm yielded favorable outcomes. In contrast, classical machine learning (ML) methods were unable to dominate the CRb-SPEA2 values. For the L class, the highest value of accuracy was recorded at 0.670, while NB among the classical machine learning methods achieved a value of 0.640. The precision and recall values were found to be 1.000, with the NB yielding a precision value of 0.885 and the SVM a recall value of 0.833. In consideration of the M class, CRb-SPEA2 once again yielded optimal accuracy and recall results, while the NB algorithm yielded the precision result of 0.522. In the H class, CRb-SPEA2 was found to be superior for all values, while other algorithms were unable to dominate the proposed method. Although classical rule-based learners could also be considered for comparison, the focus of this study is to benchmark the proposed CRb-SPEA2 framework against widely used conventional classifiers. Thus, it has been proven that the proposed rule-based evolutionary algorithm not only performs well in a competitive environment but also generates interpretable decision rules.

The findings of this study offer a powerful, data-driven alternative to traditional calendar-based spraying or purely phenological observation-based decision-making mechanisms in the fight against *Rhagoletis cerasi*, one of the most important pests threatening cherry production. The rule-based CRb-SPEA2 algorithm used in the study classified pest population density (Low, Medium, High) with high accuracy using basic pomological data such as fruit firmness, color, and acidity. The innovative aspect of the method used in this study, unlike black-box models, yielded more successful results than classical machine learning methods thanks to its explainable rules (using an if–then structure) that determine the fruit characteristics affecting pest density. In the experiments, for our first class, L, the highest accuracy value of 0.670 and precision and recall performance metrics of 1.000 were obtained with the CRb-SPEA2 algorithm. In this class, accuracy values of 0.640 and precision values of 0.885 were obtained with the classical ML method NB. In class M, a more successful result was obtained than in class L, with an accuracy of 0.739 and a recall value of 0.800. The closest result to this algorithm was given by the Naive Bayes algorithm with an accuracy of 0.522. The most successful accuracy result was obtained in class H, reaching 0.826, while the recall and precision values reached 1.000. In recent years, deep learning techniques such as Faster R-CNN and YOLOv5, and image processing algorithms have been used in pest classification. In [21], fly images from traps were examined and it was stated that 90–95% accuracy rates were achieved. Unlike these studies, the derived rules with CRb-SPEA2 provide producers and experts with the opportunity to prevent unnecessary chemical use by showing with mathematical precision at which physical stage of the fruit the risk of pests increases. The most important outcome of the study is its potential to assist decision-makers on three key issues: reducing pest residue risk, environmental sustainability, and optimizing the timing of biotechnological control.

CRb-SPEA2 is a multi-objective evolutionary rule mining algorithm that generates multiple Pareto-optimal rule solutions representing different trade-offs between Accuracy, Precision, and Recall. In contrast, classical machine learning algorithms (MLP, SVM, IBk, NB) produce a single classification model and therefore provide a single set of performance metrics. “N/A” indicates that the corresponding metric could not be computed by WEKA because no positive predictions were produced for the respective class (Table 6).

In addition to comparing the obtained results with classical machine learning methods, it is also important not to overlook the relationship of the proposed approach with other interpretable learning methods such as decision trees and fuzzy rule systems. These models also provide human-readable results, but most of them are based on single-objective optimization and deterministic models. In contrast, the proposed CRb-SPEA2 algorithm integrates evolutionary rule mining with multi-objective optimization and optimizes accuracy, precision, and recall values while also ensuring rule interpretability. Therefore, the proposed approach can be considered a bridge between performance-oriented optimization methods and interpretable rule-based models. Although decision trees (DTs) are widely used due to their transparency, they tend to develop structural complexity as the number of pomological variables increases, often leading to deep, unreadable trees that can overfit a given dataset. In contrast, CRb-SPEA2’s multi-objective structure allows for strategic pruning of the search space while maintaining a minimum rule set without sacrificing classification success. Furthermore, unlike fuzzy rule systems that require expert-led definition of membership functions, the proposed algorithm autonomously extracts clear ‘if–then’ rules directly from biological data. This autonomous feature extraction, combined with the ability to search globally across chaotic maps, guarantees that the model provides a more robust and generalized interpretation of cherry fruit fly infestation levels than standard deterministic or single-objective rule-based classifiers.

The interpretability of the proposed model was analyzed using structural complexity metrics on the obtained values. Rules with an average rule length (ARL) of less than 5 conditions are considered interpretable and accessible for human expert validation. Furthermore, the observed maximum rule length was found to be 4.66 on average, ensuring that the rules generated by the model do not exceed the cognitive processing limits of human experts. The ARL, representing the number of antecedent conditions per rule, was found to be 3.45 and is shown in Table 7. In addition, rule coverage (RC) analysis was performed, showing that the model successfully generalized across the feature space and covered 0.820 of the test data. These results demonstrate that, in addition to providing a high level of transparency, the proposed method offers a superior balance between prediction performance and practical benefit in agricultural decision-making processes compared to black-box models.

## 4. Conclusions

In this study, a classification model based on the CRb-SPEA2 algorithm was applied to create a decision mechanism based on fruit pomological data in cherry fruit fly control. The proposed method has managed to increase the prediction accuracy to over 82% at high population densities. No classical machine learning method has achieved these values. Although existing methods in the literature have high accuracy rates, they are fundamentally based on black-box mechanisms and therefore cannot provide guiding/explanatory results to decision-makers. Furthermore, these high-performance models are generally based on big data or image processing and have significant computational costs. The proposed study eliminates all these disadvantages, providing decision-makers with high performance with lower resource consumption. Overall, the greatest contribution of the proposed method to the literature is that it produces explainable results for the decision-maker by including physical parameters (fruit hardness, color, and acidity, etc.) in the model. In conclusion, this model fills a gap in the literature as a tool that generates both data-driven and biologically interpretable rules for the timing of pest control in agricultural applications. Although the proposed CRb-SPEA2 framework provides interpretable rule-based solutions with competitive classification performance, its evolutionary and iterative structure may increase computational cost when applied to larger datasets. Future studies may address this limitation by integrating hybrid optimization strategies that combine evolutionary search with local refinement techniques. In addition, sensitivity analysis of the chaotic mapping parameters and parallel or distributed implementations of the algorithm may further improve convergence speed and scalability. These directions may enable the proposed framework to be applied more efficiently in large-scale agricultural monitoring systems and real-time decision support environments. Future studies may explore hybrid strategies combining evolutionary rule mining with local search or memetic optimization techniques, as well as adaptive parameter control and parallel evolutionary computation to accelerate convergence and improve solution diversity.

## Figures and Tables

**Table 1:** Characteristics of the pomological dataset used in the experiments, including the measured fruit features, data types, and their roles in the CRb-SPEA2 classification framework.

Dataset Used	Total Samples	Classes	Samples Per Class	Features	Min Value	Max Value
Cherry fruits of the different pomological stages	381	Low/L	204	Color	1.00	5.00
		Medium/M	101	Weight	2.86	7.03
		High/H	76	Width	14.28	23.26
				Length	15.23	29.89
				StemLength	13.01	19.81
				Hardness	14.44	61.14
				Core_weight	0.60	10.0
				SCKM	12.7	17.0
				NaOH	8.83	15.48
				Acidity	0.59	1.07

**Table 2:** Confusion matrix components used to calculate classification performance metrics, including True Positive (TP), True Negative (TN), False Positive (FP), and False Negative (FN).

IF Part	THEN Part	Operation
True	True	Increase TP by 1
False	False	Increase TN by 1
True	False	Increase FP by 1
False	True	Increase FN by 1

**Table 3:** Extracted classification rules for Low (L), Medium (M), and High (H) classes generated by the CRb-SPEA2 algorithm, together with their corresponding performance metrics: Accuracy (Acc), Precision (Pre), and Recall (Rec).

Rules	Acc	Pre	Rec
IF (16.033 < width < 20.125) and (27.437 < StemLength < 54.816) and (2.265 < hardness < 6.567) THEN L	0.670	0.853	0.468
IF (3.227 < weight < 6.750) and (17.967 < length < 25.037) and (0.917 < acidity < 1.067) THEN L	0.652	1.000	0.339
IF (3.614 < weight < 5.390) and (1.059 < acidity < 1.067) THEN L	0.539	0.539	0.100
IF (2.553 < color < 3.034) and (18.558 < length < 28.505) and (17.421 < height < 18.507) and (1.725 < hardness < 8.558) and (9.279 < NaOH < 11.356) THEN M	0.739	0.500	0.033
IF (18.039 < width < 21.684) and (11.106 < NaOH < 15.465) and (0.809 < acidity < 1.061) THEN M	0.739	0.500	0.800
IF (18.511 < width < 22.013) and (9.088 < NaOH < 15.480) THEN M	0.408	0.279	0.435
IF (1.939 < color < 4.581) and (3.912 < weight < 6.412) and (14.784 < width < 23.137) and (18.003 < length < 24.551) and (15.967 < height < 18.826) and (0.374 < core_weight < 0.612) and (9.760 < NaOH < 13.742) and (0.741 < acidity < 0.998) THEN H	0.826	0.588	0.043
IF (2.469 < color < 3.617) and (4.201 < weight < 6.643) and (27.836 < StemLength < 40.043) and (0.597 < core_weight < 0.645) and (10.833 < NaOH < 12.718) and (0.632 < acidity < 0.936) THEN H	0.809	1.000	1.000
IF (14.042 < height < 19.591) and (0.942 < hardness < 2.424) THEN H	0.687	0.390	1.000

**Table 4:** Statistical performance results of the CRb-SPEA2 algorithm for all classes during the training phase, including Accuracy, Precision, and Recall values obtained from repeated experimental runs.

Classes	L			M			H		
	Acc	Pre	Rec	Acc	Pre	Rec	Acc	Pre	Rec
Min	0.489	0.534	0.042	0.398	0.257	0.014	0.308	0.214	0.038
Max	0.692	1.000	1.000	0.767	1.000	0.990	0.857	1.000	1.000
Mean	0.595	0.756	0.518	0.677	0.534	0.329	0.729	0.503	0.557
Median	0.602	0.750	0.467	0.733	0.510	0.254	0.786	0.469	0.519
Std	0.049	0.164	0.299	0.102	0.210	0.254	0.135	0.196	0.239

**Table 5:** Statistical performance results of the CRb-SPEA2 algorithm for all classes during the test phase, including Accuracy, Precision, and Recall values obtained from repeated experimental runs.

Classes	L			M			H		
	Acc	Pre	Rec	Acc	Pre	Rec	Acc	Pre	Rec
Min	0.470	0.526	0.065	0.296	0.000	0.000	0.278	0.000	0.000
Max	0.670	1.000	1.000	0.739	0.500	0.800	0.826	1.000	1.000
Mean	0.573	0.731	0.511	0.639	0.242	0.231	0.687	0.362	0.501
Median	0.565	0.706	0.403	0.683	0.250	0.133	0.709	0.333	0.478
Std	0.050	0.172	0.298	0.107	0.134	0.230	0.124	0.143	0.289

**Table 6:** Performance comparison between the CRb-SPEA2 Pareto-optimal rule solutions and classical machine learning algorithms (MLP, SVM, IBk, and Naïve Bayes) evaluated using Accuracy, Precision, and Recall metrics.

Classes	CRb-SPEA2			MLP			SVM			IBk			NB		
	Acc	Pre	Rec	Acc	Pre	Rec	Acc	Pre	Rec	Acc	Pre	Rec	Acc	Pre	Rec
L	0.652	1.000	0.355												
	0.670	0.853	0.468	0.605	0.823	0.773	0.614	0.733	0.833	0.588	0.773	0.773	0.640	0.885	0.697
	0.539	0.539	1.000												
	0.670	0.640	0.887												
M	0.739	0.500	0.100												
	0.408	0.279	0.800	0.605	0.333	0.357	0.614	0.385	0.536	0.588	0.355	0.393	0.640	0.522	0.429
	0.731	0.429	0.100												
	0.478	0.266	0.567												
H	0.826	0.588	0.435												
	0.809	1.000	0.043	0.605	0.364	0.400	0.614	N/A	0.000	0.588	0.294	0.250	0.640	0.385	0.750
	0.687	0.390	1.000												
	0.809	0.667	0.087												

**Table 7:** Quantitative interpretability metrics of the Pareto-optimal rule sets generated by the CRb-SPEA2 algorithm, evaluated using the test dataset.

Classes	L	M	H	Overall Average
Average Rule Length (ARL)	3.24 cond.	3.48 cond.	3.62 cond.	3.45 cond.
Maximum Rule Length	4 cond.	5 cond.	5 cond.	4.66 cond.
Rule Coverage (RC)	0.821	0.784	0.856	0.820
Dominant Features	Width, Hardness	Weight, NaOH	Hardness, Acidity	-

## Data Availability

The data presented in this study are available on request from the corresponding author. The data are not publicly available as the dataset used in the academic study was collected within the scope of the TÜBİTAK 1001 project numbered as 123O399 and FÜBAP project numbered as MF.24.115. Data can only be shared if the teams collecting the data consent to sharing this data with other parties.

## Abbreviations

The following abbreviations are used in this manuscript:

CRb-SPEA2	Chaotic Rule-based–Strength Pareto Evolutionary Algorithm 2
ML	Machine Learning
MLP	Multilayer Perceptron
SVM	Support Vector Machine
kNN	k-Nearest Neighbor
NB	Naïve Bayes
L, M, H	Low, Medium, High
